# 10-year experience with umbilical cord blood IgE and microbiome therapy

**DOI:** 10.1186/s13052-019-0620-3

**Published:** 2019-03-11

**Authors:** Jiří Liška, Konrad Siala, Blanka Čuláková, Václav Holeček, Štěpánka Sobotová, Josef Sýkora, František Šefrna

**Affiliations:** 1 Mulac Hospital, Newborn Department, Pilsen, Czech Republic; 2 Department of Clinical Chemistry and Hematology, Mulac Hospital, Pilsen, Czech Republic; 3 Department of Pediatrics, Charles University, Faculty of Medicine, Faculty Hospital, Pilsen, Czech Republic; 4 Department of Health Accounting and Statistics, University Hospital Pilsen, Pilsen, Czech Republic

**Keywords:** Microbiome, Allergy, Immunology, Newborn, Probiotics

## Abstract

**Background:**

The benefit of probiotics in newborn children in relation to allergy and general morbidity later in life appears to be controversial. Allergic diseases represent an increasingly important health problem worldwide in recent years. This is evident in all age groups. The occurrence of allergic illnesses also continues to rise exponentially, and thus the use of preventive and prognostic methods, particularly in children with an inherently higher risk of allergy, is gaining increased importance.

Since the advent of probiotics the effect of probiosis on immunity through alterations of composition and function of the human gut microbiome has been increasingly studied. The exact mechanisms have not yet been clearly defined.

The Academy of Sciences of the Czech Republic (The Czech Academy of Sciences has suggested that the expression of TH1 and TH2 cytokines in umbilical blood is associated with an increased risk of allergies. The counter -balance of Th1 and Th2 affect Immunoglobulin E (IgE) production and maturation of the gastrointestinal tract epithelium.

**Case presentation:**

We examined IgE levels in 3000 samples of umbilical blood taken from children born into families with a positive history of allergy in one or both parents from 2007 to 2017.

At the age of ten days, those with high IgE were given Colinfant Newborn (a lyophilized non-pathogenic strain of Escherichia.coli) for one month, three times weekly. At 15 months and three years we investigated the levels of Immunoglobulins E,A and G, and the incidence of illness and allergy.

The results revealed that allergy and high umbilical IgE is strongly linked with family history (*p* ≤ 0.001). We also detected differences in seasonality, especially with regards to pollen allergies. Eighty percent of children treated with Colinfant Newborn had significantly reduced IgE and morbidity at 13–15 months and 3 years, and furthermore without any clinical signs of allergy. Normalization of Immunoglobulins A and G was seen in 90% of treated subjects (*p* ≤ 0.001). These levels significantly correlated with an almost negligible morbidity up to 4 years of life.

Colinfant Newborn, a lyophilized strain of Esherichia coli (*E. coli*), and a normal component of intestinal flora, readily colonizes the intestinal tract. It’s long term presence significantly stimulates the production of specific and non-specific intestinal antibodies. and optimalizes immune development through tolerance. In our study Colinfant Newborn reduced the incidence of infections later in life by safely and effectively normalizing immunoglobulin levels in the majority of treated patients.

**Conclusion:**

Our study strongly suggests as positive effect of physiological *Escherichia coli* on the microbiome of newborn children as evidenced by a significantly reduced incidence of allergy and morbidity when applied early in life. These benefits appear to be strongly strain specific.

## Background

The benefit of probiotics in newborn children in relation to allergy and general morbidity later in life appears to be controversial. Allergic diseases represent an increasingly important health problem worldwide in recent years. This is evident in all age groups. The occurrence of allergic illnesses also continues to rise exponentially, and thus the use of preventive and prognostic methods, particularly in children with an inherently higher risk of allergy, is gaining increased importance. Since the advent of probiotics the effect of probiosis on immunity through alterations of composition and function of the human gut microbiome has been increasingly studied. The exact mechanisms have not yet been clearly defined. The Academy of Sciences of the Czech Republic has suggested that the expression of TH1 and TH2 cytokines in umbilical blood is associated with an increased risk of allergies. The counter -balance of Th1 and Th2 affect IgE production and maturation of the gastrointestinal tract epithelium.

## Case presentation

Allergic diseases represent an increasingly important health problem worldwide in recent years. This is evident in all age groups. The occurrence of allergic illnesses also continues to rise exponentially, and thus the use of preventive and prognostic methods, particularly in children with an inherently higher risk of allergy, is gaining increased importance.

Prevalent Th2 response supports allergy development, Th1 and Th17 responses are important for anti-infection defense but their overstatement facilitates autoimmune reactions. Probiotics are thought to promote immune system maturation.

The main objective was to evaluate the beneficial effects of early colonization with probiotic Colinfant Newborn on the immature immune system of newborns in relation to allergy and early application of the vaccine as a preventive measure against the development of allergies.

## Materials and methods

This study was carried out at a 10 bed unit of the Neonatology Department in a tertiary hospital in Pilsen, in the Czech Republic.

**Fig. 1:**
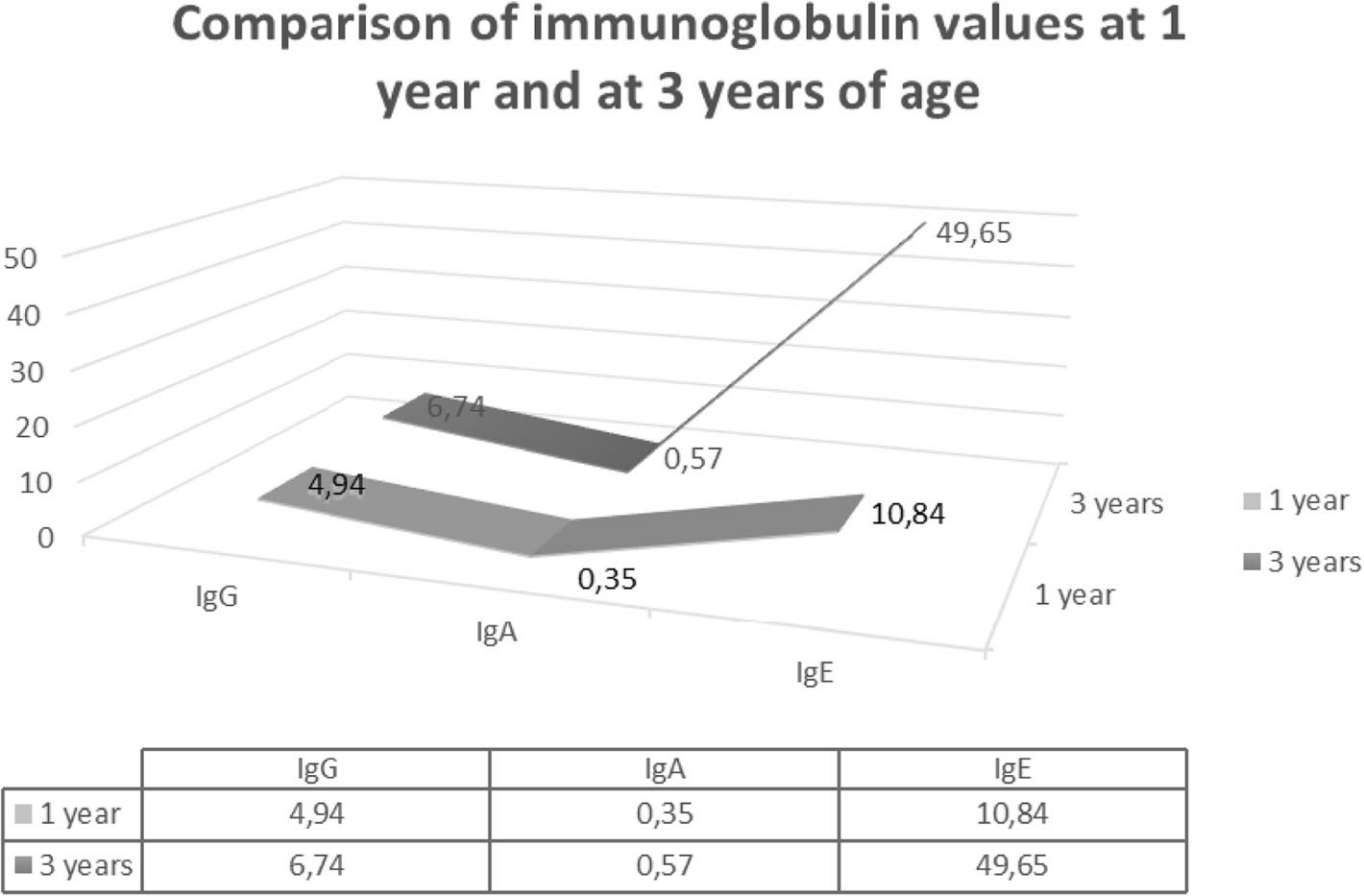
Comparison of immunoglobulin values with Colinfant at 1 and 3 years of age

**Fig. 2:**
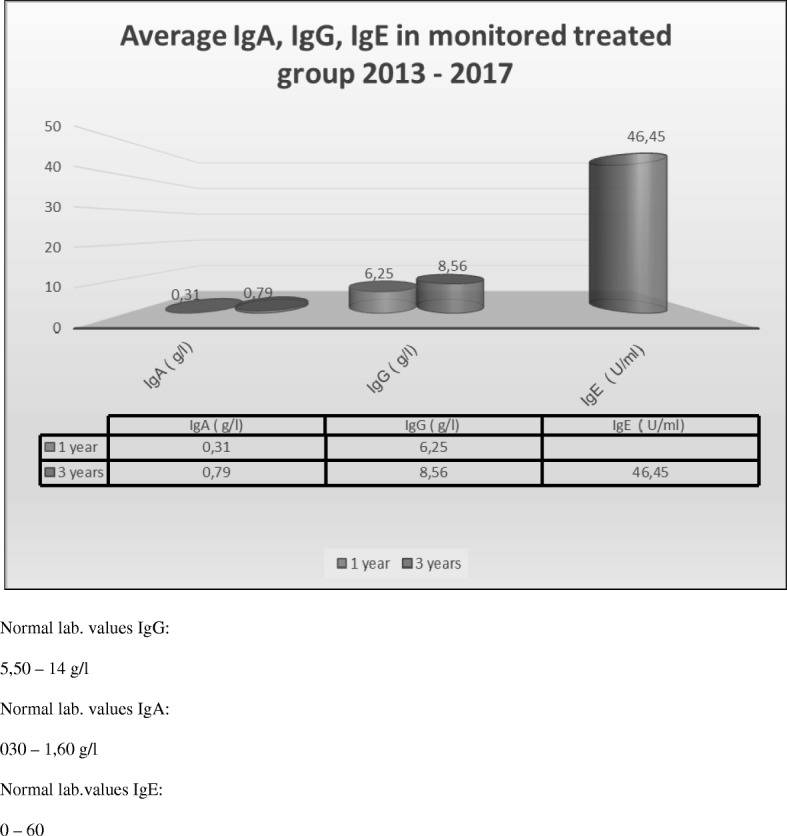
Average IgA, IgG, IgE in monitored treated group (50 ch.) in the year 2013–2017

**Fig. 3:**
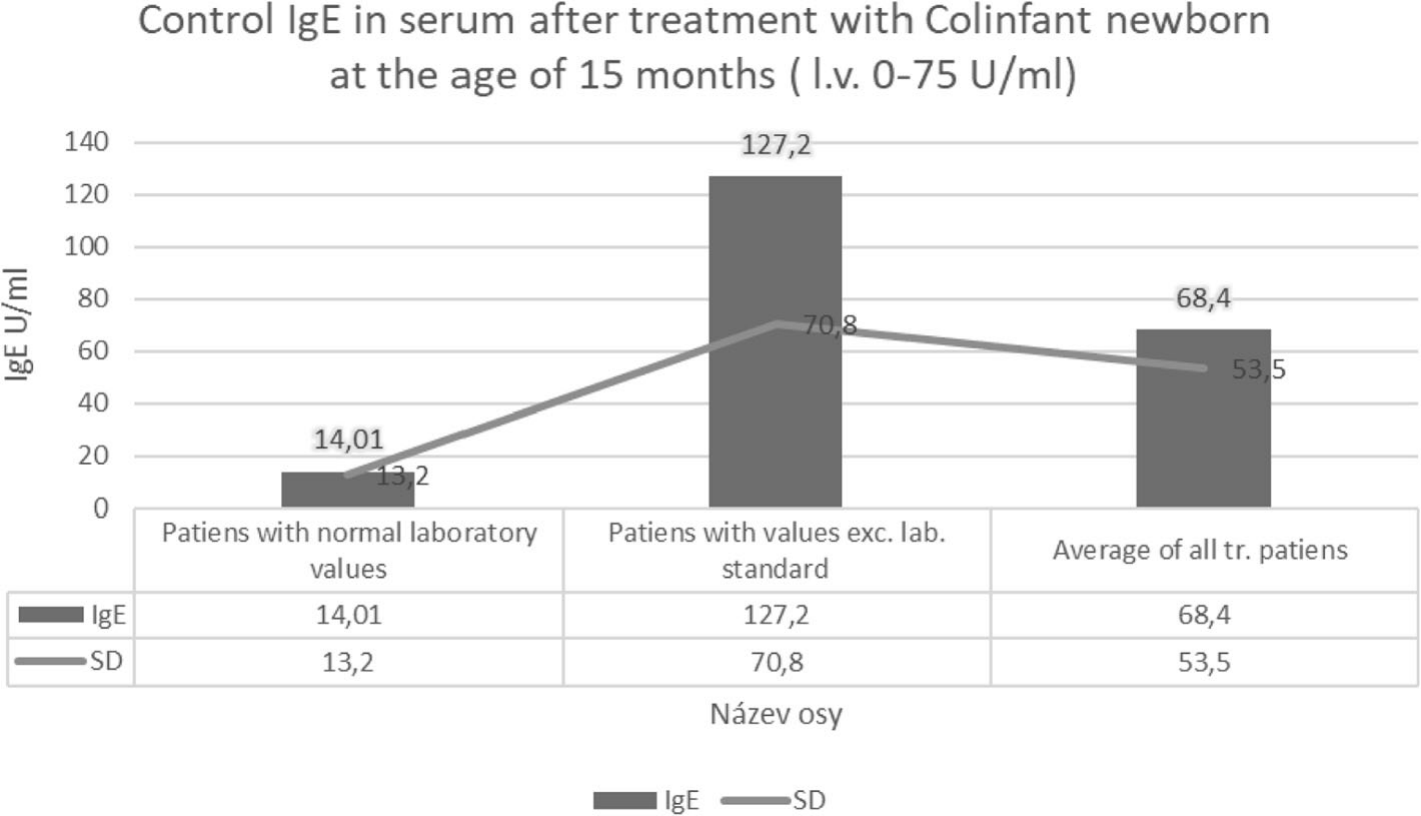
Control IgE in serum after treatment with Colinfant newborn at the age of 15 months, 2010–2015

From 2007 to 2017 IgE was measured at birth from umbilical cord blood. 2 ml of umbilical blood was collected without the use of anticoagulant agents, and analyzed using Radim ELISA kits, calibration range 0.4–1000 IU, with expected values under 5 years as less than 75 IU/ml /0–75/IU/l. Children with IgE values over 0.9 IU/ml were then included into the test group whereupon Colinfant was given from the age of 7–10 days of life as per manufacturer’s instructions. One bottle was administered three times a week for a period of 4 weeks. These test subjects were seen again 13–15 months and after three years of life whereupon we further established IgE, IgA and IgG values (Fig. 3). Determination of IgA was performed using immunoturbidimetric kits IgA K Assay Beckmann coulter on the 480 Beckman Coulter Analyzer. The calibration range was 0.2-8 g/l, with normal values up to the age of 5 years being 0.30–1.60 g/l, with expected values for children up to one month of age being 0.3–1.6 g/l. Determination of IgG was also performed using immunoturbidimetric kits IgG Beckham Coulter, on the AU 480Beckman Coulter Analyzer. The calibration range was 0.004-30 g, with expected values up to 1 year of age being 5.5–14.7 g/l, and up to the age of 15 years of age being 5.5–14.0 g/l. During both encounters all family members of each test subject were invited and questioned regarding the incidence of both general illness as well as allergic symptoms (Figs. 1 and 2).

It is important to note that umbilical IgA was measured throughout the entire study at birth. This was in order to eliminate the possibility of mixed maternal blood.

The control group comprised of subjects without a family history of allergy and with normal umbilical IgE levels. Subjects with a family history, maternal or paternal, of autoimmune disease, hormonal or on cytostatic therapy were excluded from the study. The control group for comparing data at year one, from reference values for this age, and year three which comprised healthy children being followed for secondary enuresis by a nephrologist.

### Biological material

#### Colinfant newborn – New Peroral vaccine – A probiotic

This is a live per oral vaccine containing lyophilized non-pathogenic *E. coli*. This strain is able to displace colonization of intestinal tract by pathogenic strains. It is originally patented by the Institute of Serums and Vaccines. Our source of 12 vial batches was derived from the present manufacturer, Dyntec in Terezín. Each vial contains *Escherichia coli* serotype 083: K24: H31 (minimum 0.8 × 10 CFU), and is representative of a group of bacteria that are part of normal intestinal microflora. The vaccine was registered at the State Institute for Drug Control in 1997 under registration number 59/762/97-C. Use of the strain and preparation of the vaccine were patented in 1989 under number 2645722. *E. coli*, from which the live vaccine is prepared, are a nonpathogenic strain with the above-mentioned serotype. The strain is highly selected, sensitive to common antibiotics, does not form enterotoxins and does not have a plasmid. It’s tremendous colonizing ability is ensured also by the presence of fimbriae demonstrated by hemagglutination. The strain has excellent colonizing characteristics, occupies receptors of in the intestinal mucosa, prevents adherence of pathogens and expels present enteropathogenic strains. In tests, the strain for germ-free piglets was found to be entirely non-pathogenic.

After colonization of newborn GIT, bacterial pathogens disappear from stool, throat and nose. The vaccine strain has been shown to persist in the intestinal tract of colonized newborns for 16 weeks, though it was demonstrated even after several years.

One bottle is administered three times a week for a period of 4 weeks. The administration is interrupted during application of antibiotics. It is not recommended to administer the vaccine during the acute stage of diarrheal disease*.*

### Statistical methods

Mann Whitney’s two sampled selection test., in the results was used an older named Wilcoxon unpaired test, the title adopted from English original. Nowadays is demanded Mann-Whitney’s American test. Wilcoxon unpaired test, Rank correlation-Spearman and Kendall.

At 3 years of age, there were statistically significant correlates between IgG and IgA at a significance level of 1 0/00. P level of significance (+++) – demonstrating a significant protective effect.

## Discussion

Between 2007 and 2017, 3000 umbilical cord blood samples were examined.

As far as the authors are aware, this is the longest study of it’s kind to measure IgE in umbilical cord blood.

From the entire group of newborn children with elevated IgE, 290 were subsequently treated with Colinfant Newborn. All had both case-history and laboratory examinations performed just after the first and third years of life.

Our study initially began with the aim of determining if the levels of IgE in umbilical blood are predictive of allergic disease in children by the age of 18 months and by the age of 3 years of life. We confirmed a strong predictive value of IgE as a prognostic indicator of future allergy. It is known that the immune system may be influenced prenatally in utero, at a time when allergens may pass through the placenta [1, 2]. A critical period when external factors may affect the immune system is during the first month of life [3–8, 12].

It has also been previously determined that a positive association exists between the placenta, placental microbiome and the metabolic health of children [9]. The microbiome is defined as the totality of microorganisms and their collective genetic material present in or on the human body and consists of 10–100 trillion symbiotic microbial cells, with bacterial genes exceeding those of the host genetic makeup by over a hundred times. Furthermore, on the basis of a polygenic foundation there is a possibility of sensitization of the fetus from the twentieth week of pregnancy through increased production of IgE, IL-4 and IL-5. Newborns also have an immaturely developed mucosal barrier which presents another entry point for potential allergens [3, 8, 10, 11, 17, 18, 27].

During the course of the last century, since there has been a significant reduction in wide-scale infectious and parasitic diseases, it has been proposed that the human immune system has switched into becoming more sensitive to common potential allergens.

An important role is played by chromosome 5, located on CD14, which contains an array of genes for cytokines including IL-4 [4, 12, 13].

During the first year of life the immune system matures as a result of antigen exposure, and thus begins to develop into a Th1 type profile. In atopic children however this reorganization does not appear, and the Th2 type profile continues with increased production of IL-4 and IL-5.

These play a critical role in the regulated formation of IgE. With the less than optimal IgA in newborns, there is also a deficiency of regulatory antibodies. This in turn results in increased permeability of the intestinal mucosa to potential antigens and thus leading to increasing production of IgE. This process is enhanced by a deficit of T suppressive lymphocytes - a state that is typically found in allergic diseases [15, 16, 19].

The synthesis of IgE has been found to occur from the 11th week and thus the concentration in cord blood is a reflection of it’s production. Lymphocytes carrying IgE have been found in cord blood.

The risk of sensitization in the first 6 months is significant because of low enzyme levels in the immature mucosa of the gastrointestinal tract. This immaturity confers decreased oral tolerance and relatively compromised absorption of macromolecules from the intestinal lumen [20].

Oral tolerance is the specific inhibition of immune reaction processes following previous contact of a specific antigen with regulatory cells located in the intestinal mucosa. This induction does not occur in the mucosa of the respiratory system where the same antigen infers a protective reaction [25].

It is important to enable oral tolerance during the first six months of life through active stimulation of the immune system through the application of probiotics.

It is known that the specific immune response with Th2 weighting develops from the time of delivery. The rationale for treatment with Colinfant Newborn is the redirection of immune memory of the allergic type from TH2 phenotype, before the immune response occurs to external stimuli [14].

Immunotherapy of allergic disease reduces the formation of IL-4 specific Th2 lymphocytes and increases the production of IFN-gamma specific Th1 lymphocytes.

The negative reoccurring application of Th2 lymphocytes??leads not only to?? the stimulation of IgE. Stimulation of the immune system also occurs via interaction with Toll-like receptors on dendritic cells. Probiotics are also able to support natural immunity through the production of antimicrobial peptides called defensins.

Ninety percent of children treated with Colinfant Newborn from 2005 to 2007, 2008–9, 2009–2014, 2012–2015 had significantly reduced IgE in all groups. Ten percent of children had no clinical symptoms of allergy, and yet still maintained significantly high IgE levels in their serum. We expect that this is due to the presence of regulatory antibodies. Furthermore, all of these treated groups had very low illness rates as presented in the graphs. We then further investigated the reasons for this reduced morbidity and discovered that treated children had greater IgG values when compared with controls and reference values. It is known that atopic children and those with higher morbidity rates have relatively low levels of IgA between the first and third year of life [23]. However, our treated children, in all treated groups had normal reference levels.

During of our study we have confirmed that cord IgE concentrations are predictive of allergic disease in children up to the age of three years.

The first versions of probiotics were mentioned by Mečnikov [28], who highlighted the importance of intestinal microflora and its important role in immune defense. It appears that colonization of the mucosal epithelium with a specific bacterial type has both nutritional and immunological importance. They may reduce mucosal pathogens, and reduce the number of intraepithelial regulatory T lymphocytes on the epithelial surface and thus increase their antigen presentation [28]. The ability of probiotics to influence intestinal flora has been documented in many studies [13, 21, 26, 29–37, 24].

To be included amongst probiotics they must fulfil several criteria. So far gene typization has been performed on only 3 kinds of probiotics*: E. coli* type Nissle, *E. coli* 83 Colinfant and lactobacillus casei species, Rhamnossus-lactobaciluus GG and Saccharomyces boullardi. The latter, however, is not human in origin and may not belong amongst true probiotics.

Today it is known that allergy manifests itself during the first three years with gastrointestinal symptoms, atopic eczemas and respiratory problems. The beneficial role of probiotics in atopic eczema is controversial [38, 39]. The failure of probiotics in this regard has been also published [40]. Rarely, negative effects of probiotics have occurred in immunocompromised and chronically ill children [41]. On the other hand [30] other authors have affirmed that probiotics may reduce the incidence of IgE associated eczemas in infants. The anti-infective effect of probiotics has repeatedly been attributed to the stimulation of nonspecific immunity stimulation. Fukushima suggested that the application of bifidobacteria caused significantly increased fecal IgA level and anti-poliovirus IgA [22]. This increase may indicate a stronger resilience against GIT infections.

The possible effect of probiotics may be due to alterations in intestinal permeability, stimulation of immune response to pathogens, the increased production of secretory IgA, IgG, IgM, and the anti-inflammatory cytokines interleukin-10 and TGF beta [42].

Other studies claimed prevention of enterocolitis in very low birth weight children by the use of probiotics [43, 44]. On the other hand, some authors have hypothesized a protective effect of early introduction of cow’s milk due to protection against IgE [45].

When breast feeding is not possible, international associations concerned with nutrition recommend hydrolyzed or partially hydrolyzed infant formulae with low allergenicity, up to the age of 4 months. The previous suggestions of excluding allergens in the nutrition of children up to the age of six months have been sidelined. In our cohort of non-breastfed patients, we recommended the exclusive use of hypoallergenic HA BEBA formula.

Authors have agreed the most intensive colonization happens during the first month of life and progressively decreases around the first year of life and remains at a constant into adulthood [46]. On the other hand, other studies have demonstrated that the application of probiotics benefitted primarily children with IgE based allergy [7].

The production of IL-10 and TGF leads to the activation of T lymphocytes and induces oral tolerance. Pathogenic microorganisms activate T lymphocytes and their cellular response. Secretory IgA antibodies, part of the humoral immune reaction, prevent the penetration of antigens from the intestinal lumen.

The possibility of positively affecting gastrointestinal flora with probiotics has been discussed in many works [33, 47–51]. The rationale for treatment with probiotics lies in redetermining the immune response from Th2 to the Th1 phenotype before the immune response reacts to the antigens in the immediate environment. The immune system in early neonatal life lacks prompt contact with microbes and allergens that are important for the stimulation and regulation of a healthy and functional immune system [52].

From our study it is evident that there are clear benefits in early colonization of strain specific probiotics in children that may be considered at higher risk of morbidity and allergy in later life.

## Conclusion

Immunoglobulin values changed over time, between the 1st-3rd years of life with a significance level of 1 0/00.

IgG treated children increased in median from 4.55 to 6.87.

IgA treated children increased in median from 0.26 to 0.62.

IgE treated children increased in median only from 8.87 to 17.09. The values remained completely within the standard. P+++ statistical significance was reached at the level of 1 promile, i.e. a significant protective effect, increased IgE and IgA. A significant decrease in IgE in line with the decreased illness rates in these patients. The development in time at controls was not tested for a small number of complete pairs of measurements.

### Correlation of variables in controls

At 1 year of age in treated subjects, there were statistically significant correlates between IgG and IgA at a significance level of 1 0/00. Correlation was positive and directly proportional.

At 1 year of age, the umbilical IgE correlated with all serum immunoglobulins, and most with IgG and IgA (Fig. 3).

P level of significance at 1 promile (+++) and 1 0/0 and reflects the positive clinical and laboratory status of the subjects. At 3 years of age, there were statistically significant correlates between IgG and IgA at a significance level of 1 0/00. P level of significance (+++) – demonstrating a significant protective effect.

Statistical evaluation of treated groups 2012–2015 demonstrated significant difference between treated and control groups in IgA at year one and IgA at three years and IgE at three years at P level of significance (+++).

At 1 year of age in 6 patients, there were statistically significantly correlates between IgG and IgA at a significance level of 1 promile correlation was positive and directly proportional.

At 1 year of year of age umbilical IgE correlates with all serum immunoglobulins the most with IgG and IgA.

P level of significance at 1 promile /+++/and 1% /++/and reflects the positively clinical and laboratory status of the children.

At 3 years of age, there were statistically significant correlates between IgG and IgA at a significance level of 1promile.

IgE in the treated children did not statistically significantly differ from the reference values for healthy children. 95% of treated children by the age of three years were minimally ill. The use of antibiotics in these children was exceptionally rare with the vast majority of treated children had never been treated with antibiotics.

## Abbreviations

GIT: gastrointestinal tract
IFN: IFN Interferon Gama
IgA: IgA immunoglobulin
IgE: IgE immunoglobulin
IgG: IgG immunoglobulin
IL4: IL4 Interleukin 4
TGFβ: TGF Beta cytokinin
Th1: Th1 lymphocytes
Th2: Th2 lymphocytes
